# The significance of Stanniocalcin 2 in malignancies and mechanisms

**DOI:** 10.1080/21655979.2021.1977551

**Published:** 2021-10-06

**Authors:** Shasha Li, Qian Huang, Dongliang Li, Lizhi Lv, Yi Li, Zhixian Wu

**Affiliations:** a Department of Hepatobiliary Disease, Dongfang Hospital, Xiamen University, Fuzhou, China; b Department of Hepatobiliary Disease, Fuzong Clinical College, Fujian Medical University, Fuzhou, China; c Department of Oncology, 920th Hospital of Joint Logistics Support Force, Kunming, China

**Keywords:** Cancer, metabolism, phosphoprotein, Stanniocalcin 2

## Abstract

Human stanniocalcin 2 (STC2) is an ortholog of fish stanniocalcins (STCs) and is widely expressed in various organs and tissues. The gene is localized on chromosome 5q33 or 5q35. STC2 has been implicated in glucose homeostasis and phosphorus metabolism. It is also reported to be implicated in various malignancies. STC2 was found to be implicated in breast cancer and gynecologic cancers, suggesting hormone-specific or -dependent activities in these malignancies. Moreover, it was reported to be involved in gastrointestinal tumors, including esophageal, gastric, colorectal, and liver cancers, and respiratory cancers, including laryngeal and lung cancers. It also influenced renal carcinoma and prostate cancer. Notably, as a secreted phosphoprotein, STC2 was detectable in serum and possessed promising predictive value in several malignancies. This review aims to improve the understanding of the role of STC2 in patient diagnosis and prognosis, and tumor development and progression, as well as the mechanisms involved.

## 1. Introduction

**Figure 1.**
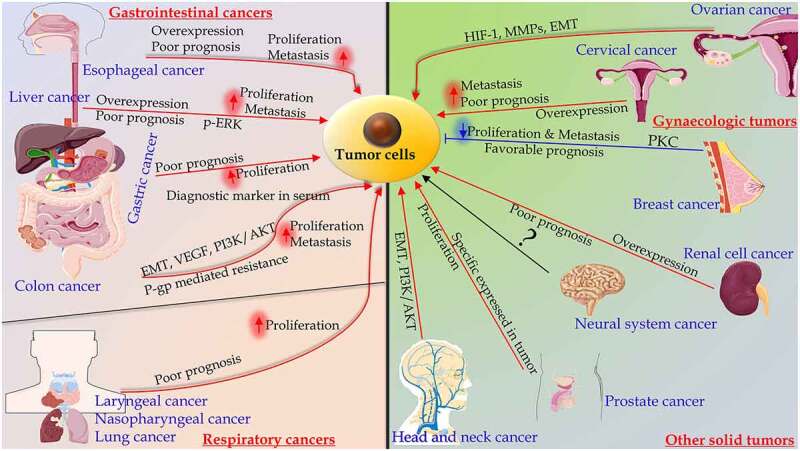
The significance of Stanniocalcin 2 in malignancies and mechanisms. Among gastrointestinal cancers, STC2 is overexpressed in esophageal, liver, gastric and colon cancers, which is associated with poor prognosis, proliferation and metastasis. In gynecologic cancers, such as ovarian cancer, STC2 plays an important role in migration and invasion, which is regulated by distinct signaling pathways. The overexpression of STC2 in cervical cancer was correlated with the poor prognosis. In breast cancer, the overexpression of STC2 partially inhibited EMT by protein kinase C (PKC)/Claudin-1-mediated signaling pathway. In respiratory cancers, including laryngeal, nasopharyngeal, and lung cancers, the overexpression of STC2 predicts poor prognosis, and improves proliferation. The levels of STC2 mRNA and protein are increased in renal cell carcinoma. STC2 is overexpressed in castration-resistant prostate cancer. The overexpression of STC2 may promote head and neck squamous cell carcinoma. STC2 may serve as a useful biomarker in neural system carcinoma

Stanniocalcin 2 (STC2) was identified in mouse and human while searching for sequences similar to Stanniocalcin 1 [1,2]. Both STC1 and STC2 have orthologs in teleost fish. STC1 is implicated in the regulation of calcium metabolism. In mammals, STC2 is widely expressed in many tissues including kidney, ovary, bone, neurones, and muscle, with a role in mineral homeostasis and tumor promotion [3]. The altered expression of STC2 has been observed in numerous cancers and possessed predictive value for patient survival [2–10] (Figure 1). There is no comprehensive review on the role of STC2 to clarify its functions and potential in various tumor context. Therefore, a thorough understanding of the role of STC2 in tumor development and progression is essential to facilitate future studies of this promising marker.

## 2. Identification and regulation of *STC2* gene

### 2.1 Identification of STC2 gene

Stanniocalcin 1 (STC1) is derived from the corpuscle of Stannius (CS), a kidney-related endocrine gland [11] with a role in regulating calcium and phosphorus metabolism [12,13]. The human ortholog of fish *STC1*, is associated with the immortalization of cells determined by mRNA differential expression of genes [1]. Chang et al. identified *STC2*, a *STC1* paralog while searching for related sequences in an expressed sequence tag (EST) database [14–16]. Ishibashi et al. cloned STC2 from human osteosarcoma cDNA library, whereas Chang et al. identified, cloned and characterized it from both human and mouse [14,16]. The human *STC2* cDNA encodes a polypeptide of 302 amino acids, which is 34% identical to human STC1 and eel STC [16]. STC2 is similar to eel STC and STC1 at the N-terminus, with 40% identity at amino acid residues 41–160 [16]. STC2 has 15 histidine residues, twice as much as found in STC1 and 5-fold as much compared to eel STC. Furthermore, four such structures cluster at the C-terminus of STC2 and may function in the transition of metal [17]. Although mammalian STC2 is not universally expressed, it is identified in a wide variety of tissues, including endocrine glands and hormone responsive organs [15].

### 2.2 Regulation of STC2 gene

The *STC2* gene has been mapped to human chromosome 5q33 or 5q35 [17,18], containing four exons [16]. This finding excludes *STC2* as a candidate gene for autosomal dominant hypophosphatemic rickets, a phosphate wasting disease that was previously localized to chromosome 12p13 [18]. The exon-intron boundaries are completely conserved between *STC2* and *STC1*, indicating common ancestry. In contrast to *STC1*, no CAG repeats were found in the 5` or 3` UTR of *STC2* [16]. Very few studies have explored genetic regulation of STC2. DiMattia’s laboratory identified that estrogen, progesterone, and retinoic acid receptors played critical role in the regulation of STC2 in human breast carcinoma cell lines T-47D and MCF7 [19].

## 3. Secretory property and physiological role of STC2

The study by Jellinek et al. stated that although STC2 could not be detected in cell lysates, it was present in conditioned media, suggesting constitutive processing and secretion of the protein [20]. Accumulating evidence indicates that mammalian STC2 is secreted as a phosphoprotein. Unfortunately, the role of this phosphorylation is still unknown. It can only be inferred from the regulatory effects of phosphorylation on other secreted phosphoproteins that it is probably a vital modification for modulating the structure, half-life, and the physiological activity of STC2 [20]. In addition to secretion by normal tissues, STC2 is also secreted by a human fibrosarcoma cell line [20]. It is phosphorylated by casein kinase II (CK2) in vitro and by an ectoprotein kinase in vivo, on a single serine between Ser285 and Ser298 [20]. More studies regarding the significance of secretory STC2 in cancer will be reviewed in subsequent section.

One of the essential physiological roles of STC2 is involving metabolism of calcium and phosphorus. The culture medium of CHO cells transfected with STC2 inhibited the transcriptional activity of kidney type II sodium-phosphate co-transporters, which is opposite effect to that of STC1 on the sodium-phosphate co-transporter [16]. A reduction in phosphorus uptake was observed in opossum kidney cells incubated in conditioned medium of STC2-transfected CHO cells for 2 days [16]. In transgenic mice, STC2 served as a growth inhibitor similar to STC1, acting on bones and skeletal muscle [21]. However, the levels of serum Ca^2+^ levels did not alter, and growth retardation was not associated with the levels of growth hormone. Takei et al. demonstrated that STC2 was positively and negatively controlled by 1, 25(OH)_2_D_3_ and pituitary thyroid hormone (PTH) in renal proximal tubular cells [22].

Another important function of STC2 is implicating osteoblast differentiation. Several studies revealed the effect of STC2 on osteoblast differentiation is exerted via a vitamin K2 isoform, menaquinone-4 targeted growth differentiation factor 15 and STC2 via protein kinase A pathway [23]. During the differentiation process of skeletal mesenchymal stem cells to osteoblasts, STC2 is one of the secretome proteins in an autocrine role [24]. Additionally, STC2 is upregulated during the osteoblast differentiation process of MC3T3-E1, an osteoblast precursor cell line [25]. The silencing of STC2 impeded osteoblast differentiation and mineralization, and down-regulated the expression of relevant genes including runt‑related transcription factor 2 (RUNX2, a key transcription factor associated with osteoblast differentiation), collagen type I α 1 chain (COL1A1, a component of type I collagen), osterix, and osteocalcin [25]. The overexpression of STC2 activated the extracellular signal‑regulated kinase 1/2 (ERK1/2), whereas the inhibition of ERK phosphorylation reduced the osteoblast differentiation of MC3T3‑E1 cells overexpressing STC2 [25].

STC2 was found in pancreatic alpha cells, suggesting that STC2 may be implicated in glucose homeostasis [17]. Interestingly, STC2 bound with heme oxygenase 1 to form a complex, thereby participating in the degradation process of heme [26]. The binding site for SCT2 was located to amino acids 181–200 and a regulatory motif of heme [26].

## 4. STC2 in breast cancer and gynecologic cancers

### 4.1 Breast cancer

Interestingly, favorable effects of STC2 expression in breast cancer were reported in several studies [27–29]. STC2 expression was found to be higher in breast cancer patients with a predisposition toward late recurrence than in those with early metastases and relapses [30]. STC2 may serve as a survival factor for breast cancer cells, contributing to tumor dormancy [30]. Esseghir et al. examined the expression of five genes including probes against thrombospondin 3, insulin-like growth factor binding protein 7, tumor rejection antigen 1, STC2, and netrin 4 in a tissue microarray containing 245 invasive breast tumors from women, which were found to be associated with a prolonged disease-free survival [31]. Only a few studies reported negative effects of STC2 on breast cancer [30]. Jiang et al. demonstrated that STC2 gene probably promotes the development and metastasis of breast cancer by interacting with estrogen and ER by analyzing its expression in 50 cases of breast cancer tissues. The study only included a small size of samples and no cellular mechanisms were explored.

The implication between STC2 and hormone were also investigated. The level of STC2 was increased as a hormone-responsive gene in the normal mammary gland of mice during puberty [32]. In human breast cancer, STC2 and estrogen receptor alpha were co-expressed, suggesting that STC2 exerted a positive prognostic role in estrogen receptor-associated breast cancer [33]. Interestingly, the expression of STC2 was distinct between inflammatory and non-inflammatory estrogen receptor-positive breast cancer patients as assessed by metagene analysis [33]. Bouras et al. demonstrated that estradiol increased the expression of STC2 in breast cancer cells, and estradiol antibody reversed the effect [29]. In estrogen-positive and -negative breast cancer, the mRNA levels of STC2 were correlated with estrogen receptor mRNA and protein levels. In addition to estradiol, progesterone and retinol acid regulated the expression of STC2 in multiple breast cancer cell lines [29]. The hormonal regulation of STC2 expression was associated with the transcriptional activity; however, it did not bind directly to the promoter region [29].

Several studies sought to explain the results by investigating the cellular mechanisms and relevant signaling pathways by which STC2 functioned in breast cancer. Hou et al. showed that 231HM, a breast cancer cell line, exhibited high motility, fibroblast morphology, enhanced cell migration, and invasion following silencing of STC2, and vice versa [7]. The overexpression of STC2 partially inhibited EMT by protein kinase C (PKC)/Claudin-1-mediated signaling pathway [7]. Santa et al. found that STC2 was a downstream target of estrogen, progesterone and retinoic acid signaling pathways, and functioned in a paracrine/autocrine fashion to reduce cell proliferation [31]. These findings suggest that STC2 mediates its effects through different signaling pathways dependent on the tumor context, possibly through dysregulation of hormone-dependent or calcium and phosphate-dependent signaling.

### 4.2 Ovarian cancer

The STC2 expression was significantly correlated with tumor grade and histological type, and was inversely correlated with patient survival, suggestive of a potential predictor of patient prognosis [4]. Under hypoxic conditions, it increased the migration and invasion of ovarian cancer cells and directly regulated the transcription of high-motility genome, which was essential for the epithelial-to-mesenchymal transition (EMT) [4]. Law et al. showed that the overexpression of STC2 increased the expression of N-cadherin and vimentin, and decreased the expression of E-cadherin [5]. The cells stably transfected with STC2 showed a high degree of motility, fibroblast morphology, and a high degree of invasiveness, which might be associated with increased expression of matrix metalloproteinases (MMPs) 2 and 9 [5]. Another study by Law et al. demonstrated that *STC2* was the target gene of hypoxia inducible factor 1 (HIF-1) and regulated ovarian cancer cell proliferation [6]. Two HIF-1 binding sites were identified on the *STC2* promoter sequence [6]. Bucknovich et al. also reported that STC2 was highly expressed in ovarian cancer, and coupled with other molecules as tumor vascular markers, facilitated the diagnosis of ovarian cancer [32]. An interesting study by Law et al. showed that the expression of STC2 was low in ovarian cancer cells (SKOV3, OVCAR3 and CaOV3) and related to CPG island promoter hypermethylation [33]. CPG dinucleotides in the promoter region of normal human ovarian cells were not methylated and had high basal STC2 levels. The CPG island promoter hypermethylation abrogated the expression of HIF-1-mediated STC2 [33]. Therefore, the expression of STC2 was epigenetically inactivated in several ovarian cancer cells, which suggests that appropriate selection of cell lines is critical in STC2 research in ovarian cancer. In another large cohort study by Wu et al., the results showed that STC2 was inversely correlated with patient survival, and enhanced cell migration and invasion, which was regulated by high-mobility gene group A2 [4]. Taken together, STC2 plays an important role in ovarian cancer and is regulated by distinct signaling pathways, which may be dependent on cancer subtypes. Further studies are warranted to elucidate the function of STC2 in ovarian cancer.

### 4.3 Cervical cancer

The expression of STC2 in cervical cancer tissues and cell lines was upregulated, and this was correlated positively with cell proliferation [8]. In cisplatin-resistant cervical cancer cells, the expression of STC2 was significantly increased. Thus, the modulation of STC2 expression regulated cell survival, apoptosis, and cisplatin resistance [8]. The underlying mechanism was associated with the regulation of STC2 with respect to the MAPK pathway [8]. Shen et al. reported that the expression of STC2 was significantly higher in tumors of cervical cancer patients than that in normal cervical tissues, which was negatively correlated with the overall survival (OS) rate after radiotherapy [19]. An increased expression of STC2 was also associated with lymph node metastasis [19]. Therefore, STC2 may be a prognostic marker in cervical cancer patients undergoing radiotherapy.

## 5. STC2 in gastrointestinal cancer

### 5.1 Oral and esophageal cancers

Kashyap et al. found that STC2 was expressed in 94% of the clinical samples of esophageal cancer [9]. Moreover, gene microarray analysis displayed the overexpression of STC2 in esophageal squamous-cell cancer [19]. The STC2 expression was related to lymphatic metastasis, lymph node invasion, and distant metastasis. The STC2-positive patients showed a poor 5-year survival rate than the negative patients. Transfection with STC2 increased the cancer cell proliferation and invasiveness [19]. Ferreira do Carmo et al. found that high expression of STC2 was significantly associated with poor disease-specific survival and high rate of recurrence. Knockdown of STC2 in oral squamous cell carcinoma cells attenuated proliferation, migration and invasiveness while increased apoptotic rates [34]. These findings suggest the potential of STC2 in development of novel therapeutics and predictive approaches in oral and esophageal cancers.

### 5.2 Gastric cancer

Several studies have revealed the clinical significance of STC2 in gastric cancer [35,36]. Yokobori et al. showed that STC2 was highly expressed in gastric cancer tissues [35]. The high levels of STC2 in gastric tissues were associated with elevated lymphatic metastasis and venous invasion. STC2 served as an independent prognostic factor for gastric cancer patients. Furthermore, knocking down STC2 in gastric cancer cell line reduced the cell proliferation [35]. Ke et al. also found that STC2 was significantly up-regulated in gastric cancer and was negatively regulated by miR-1-3p. Conversely, the upregulation of STC2 weakened the inhibitory effect of miR-1-3p in gastric cancer [37].

In addition to tissue expression, STC2 was explored in sera from gastric cancer patients. Wang et al. examined the STC2 level in peripheral blood of patients with gastric cancer and demonstrated satisfactory diagnostic potential of STC2 to differentiate from healthy controls by using receiver operating characteristics curves [38]. Similar results were reported in another study on gastric cancer by Fang et al. [39]. However, these studies only included a small size of patient samples and the significance of serum STC2 needs to be verified in large-scale studies and more tumors.

### 5.3 Colorectal cancer

Studies on STC2 in the forementioned gastrointestinal tumors were barely involving in vitro mechanisms. Nevertheless, it was encouraging in colon cancer. Several studies showed similar results that STC2 levels were higher in colorectal cancer (CRC) tissues than normal tissues, which were related to tumor size, histological grade, lymph node metastasis, lymphatic invasion, and tumor depth [40,41]. Notably, the serum STC2 levels were also examined and found to be correlated with the pathological grade and poor patient survival, suggesting the potential of STC2 as a promising serum biomarker [42]. Further study revealed that STC2 promoted EMT and colorectal cancer migration [42]. Zhang et al. utilized both the online databases and clinical samples to demonstrate the role of STC2 in CRC progression and prognosis, and the potential in survival prediction [43]. Further in vivo studies have explored the cellular signaling pathways involved. The activation of ERK/MEK and PI3K/AKT signaling pathways was implicated in STC2-induced EMT and CRC progression [42]. In addition, Li et al. demonstrated that STC2 participated in the development and progression of CRC by activating the Wnt/β‑catenin signaling pathway [44]. STC2 is also a target of regulation by other factors. Li et al. provided evidence that the transcription factor Sp1 was essential for the overexpression of STC2 in colon cancer through the activation of promoter [45]. Different pathways identified may result from distinct cell lines employed or subtypes of cancer.

STC2 was also implicated in the development of chemoresistance [46]. Monoclonal antibody against vascular epithelial growth factor (VEGF) is one of the effective treatments for CRC, which is often interfered by anti-VEGF resistance [46]. Microarray analysis indicated that STC2 was significantly up-regulated upon the development of resistance [47]. Additionally, STC2 was found to play a role in the chemoresistance to oxaliplatin [48]. Knocking down STC2 sensitized the CRC cells to oxaliplatin, whereas transfection with STC2 in chemonaïve CRC cells induced oxaliplatin resistance. The expression of P-glycoprotein (P-gp) expression was elevated by STC2, and inhibition of the PI3K/AKT signaling pathway reduced the expression of P-gp, which indicated that oxaliplatin resistance was related to STC2-induced P-gp expression via the PI3K/Akt pathway [48]. Taken together, STC2 possessed remarkable clinical significance in CRC patients and induced EMT and chemoresistance via several potential pathways. More studies are needed to prove that STC2 is a useful therapeutic target.

### 5.4 Liver cancer

Wang et al. showed that STC2 was upregulated in hepatocellular carcinoma (HCC) and correlated with the tumor size and multiplicity of HCC [10]. The aberrant expression of STC2 promoted cancer cell growth, invasion, and colony formation while silencing STC2 delayed the cell cycle in G0/G1 phase. Further study revealed that STC2 regulated cyclin D1 and activated ERK 1/2 [10]. Wu et al. demonstrated that methyl methanesulfonate (MMS) and UV-sensitive gene clone 81 (Mus81, a protein involved in the recognition and/or processing of certain types of DNA damage caused by UV and MMS) and STC2 both had cancer-promoting roles in HCC, which were co-expressed in HCC tissues [49]. Knocking down *Mus81* inhibited cell proliferation and increased cell apoptosis. On the other hand, the down-regulation of STC2 and transfection with STC2 restored cell growth in Mus81-depleted cells, thereby suggesting that Mus81 promoted HCC via STC2 regulation [49]. Guo et al. found that miR-485-5p, a hotspot miRNA, inhibited cancer cell growth via targeting STC2 [50]. The overexpression of miR-485-5p reduced the level of STC2, but not of STC2 with mutant 3ʹ-UTR. Consistently, the inhibition of miR-485-5p decreased the levels STC2. Wang et al. showed that both STC2 mRNA and protein expression were correlated with clinicopathological parameters and predicted patient survival by analyzing HCC and adjacent tissues from 200 patients. In liver cancer, it was suggested that STC2 interacted with various factors to exert effects and more studies are needed to fully understand the role of STC2 in these complex networks.

## 6. STC2 in respiratory cancers

### 6.1 Laryngeal cancer

STC2 is highly expressed in laryngeal cancer but not in normal tissues [51]. The overexpression of STC2 was correlated with clinical stage, tumor location, and histological grade. The STC2-positive group showed a poorer rate of survival than the STC2-negative group. Zhou et al. revealed that the levels of STC1 and STC2 in peripheral blood of laryngeal cancer patients were significantly higher than those in healthy volunteers [52]. The expression of STC2 in tumor tissues was related to thyroid cartilage invasion, T-stage, lymphatic metastasis, clinical stage, and pathological differentiation. STC2 was an independent prognostic factor for the OS of patients with laryngeal squamous cell carcinoma [52].

### 6.2 Nasopharyngeal cancer

Lin et al. showed that STC2 was expressed in a majority of the clinical samples of nasopharyngeal carcinoma [53]. STC2-positive patients had significantly lower OS than the STC2-negative patients. Progression-free survival and metastasis survival were also declined. In patients undergoing radiotherapy, STC2 overexpression predicted a high risk of residual tumors [53]. In vitro, He et al. further demonstrated that STC2 promoted survival and metastasis of post-radiation nasopharyngeal carcinomas cells [54]. Therefore, STC2 possesses predictive value in nasopharyngeal cancer patients and may serve as a therapeutic target to overcome radiation resistance and metastasis. Nevertheless, the downstream pathways still need to be elucidated. Li et al. employed nasopharyngeal carcinoma cell lines to explore the pathways for dysregulation of STC2 and found ITGB2/FAK/SOX6 axis was involved. The evidence level of the study was inadequate due to a small sample size.

### 6.3 Lung cancer

The overexpression of STC2 was observed in lung cancer cells, and knockdown of STC2 suppressed the growth, colony formation, invasion, and metastasis of cancer cells [55]. The overexpression of STC2 in lung cancer tissues was also observed. Additionally, STC2 exerted a protective effect on the redox system of lung cancer [55]. Liu et al. found that STC2 overexpression induced acquired resistance to epidermal growth factor receptor tyrosine kinase inhibitors via the STC2-JUN-AXL-ERK signaling pathway. These studies indicate that STC2 also plays a tumor-promoting role in lung cancer and is involved in treatment resistance via a pathway that is not revealed in other cancers.

## 7. STC2 in other solid tumors

### 7.1 Renal cell carcinoma

The level of *STC2* mRNA and protein were increased in the tissues of renal cell carcinoma (RCC) [56]. The staining location in RCC was different from that in normal renal tissues [56]. STC2 expression was observed in clear cells, chromophores, and papillary RCC. Survival analysis suggested that high levels of STC2 were correlated with short survival. STC2 even served as a risk factor for RCC without metastasis [56].

### 7.2 Prostate cancer

Microarray analysis indicated that STC2 was an overexpressed gene in castration-resistant prostate cancer (PC) [57]. Increased levels of *STC2* mRNA and protein were detected specifically in castration-resistant PC cells and aggressive castration-naïve PCs with high Gleason scores [57]. STC2 was not expressed in normal prostate tissues and indolent castration-naïve prostate cancers. The knockdown of STC2 reduced the expression, and vice-versa, the overexpression promoted the cancer cell growth and proliferation [57]. Therefore, STC2 might serve as a novel biomarker for the diagnosis and treatments of aggressive prostate cancer. Nevertheless, high-level evidence is still lacking to verify the potential of STC2 in prostate cancer.

### 7.3 Neural system carcinoma

Under hypoxic conditions, enriched STC2 was identified from the exosome and soluble fractions of glioma cell lines [58]. It was highly correlated with glioma grade in human patients. An increased cell migration was induced by STC2 in a hypoxic manner. Conversely, STC2 reduced cell growth and increased the apoptosis in human neuroblastoma cell lines [59]. However, it promoted cell invasion, upregulated the activity of MMP2, and increased the cell migration to disrupt the blood vessels, giving rise to massively bleeding tumors [59].

### 7.4 Head and neck squamous cell carcinoma

In head and neck squamous cell carcinoma, the overexpression of STC2 suppressed cell apoptosis, promoted cell proliferation, migration, invasion, and delayed the cell cycle at the G1/S phase; all these phenomena could be reversed by knocking down STC2 [60]. STC2-enhanced cancer metastasis was associated with an increase in vimentin and decrease in E-cadherin mediated by Snail [60]. The analysis of tumor samples from patients with lymph node metastasis revealed that the expression of STC2 was correlated with the levels of AKT and Snail [60], thereby suggesting that STC2 may promote head and neck squamous cell carcinoma via PI3K/AKT/Snail pathway. Li et al. showed that STC2 was regulated by homeobox transcript antisense RNA in cell lines by sponging microRNA-206 [61]. In head and neck squamous cell carcinoma, STC2 promotes tumor and its correlationship with clinical parameters needs to be further analyzed.

## 8. STC2: a universal serum tumor marker?

In addition to expression in cancer tissues, STC2 was detected in patient sera from several cancers including gastric cancer [38], colorectal cancer [42], and laryngeal cancer [52]. Although it was unclear whether STC2 existed in the serum of glioma patients, it was secreted in the conditional medium of tumor cell lines [58]. A human fibrosarcoma cell line also secreted STC2 [20]. Nonetheless, whether STC2 is competent as a serum marker in massive epithelial and non-epithelial tumors, whether the secreted STC2 exerts autocrine or paracrine effects, or acts only as a nonspecific metabolite, and whether the secretion is valuable, needs to be elucidated further with respect to its receptor. In the future studies, serum STC2 from various malignancies can be compared to determine in which cancer it is elevated remarkably and stably, that is to say, great potential in diagnosis.

## 9. Conclusions

STC2 has been found to be implicated in the tumor development and progression in several malignancies and appears to be a promising marker for disease severity and patient prognosis. Uniquely, STC2 exerted negative effects on tumor development and progression in breast cancer, which may be caused by complexed hormone-dependent mechanisms. Based on the pivotal findings, further studies are warranted to provide insights into the role of STC2 in cancer biology including metastasis and tumor angiogenesis, and potential as biomarkers and therapeutic targets.

## Data Availability

The main document contains all the data

## Highlights

• In mammals, STC2 is widely expressed in many tissues and plays a role in mineral homeostasis.

• STC2 is overexpressed in numerous tumors and is associated with disease severity and prognosis.

• Serum STC2 possesses diagnostic value in several cancers, especially gastric cancer.

## Abbreviations

cDNA: complementary DNA; COL1A1: collagen type I α 1 chain; CRC: colorectal cancer ; EMT: epithelial-to-mesenchymal; ERK1/2: extracellular signal-regulated kinase 1/2; HCC: hepatocellular carcinoma; HIF-1: hypoxia inducible factor 1; MMPs: metalloproteinases; Mus81: methane sulfonate and UV-sensitive gene clone 81; OS: overall survival; PC: prostate cancer; PTH: pituitary thyroid hormone; P-gp: P-glycoprotein; RCC: renal cell carcinoma; RUNX2: runt-related transcription factor 2; STC: Stanniocalcin; STC2: Stanniocalcin 2; VEGF: vascular epithelial growth factor
